# Inhibitory Influence of *Panax notoginseng* Saponins on Aspirin Hydrolysis in Human Intestinal Caco-2 Cells

**DOI:** 10.3390/molecules23020455

**Published:** 2018-02-18

**Authors:** Zongxi Sun, Yali Wu, Bing Yang, Baochen Zhu, Shaonan Hu, Yang Lu, Bo Zhao, Shouying Du

**Affiliations:** School of Chinese Materia Medica, Beijing University of Chinese Medicine, Beijing 100029, China; zongxisun@163.com (Z.S.); wuyali1993@163.com (Y.W.); yangbing1028@163.com (B.Y.); zbcbock123@sina.com (B.Z.); hushaonan8980@126.com (S.H.); zhaobowua@163.com (B.Z.)

**Keywords:** *Panax notoginseng* saponins, aspirin, Caco-2 cells, intestinal absorption, carboxylesterase

## Abstract

Herb-drug interactions are important safety concerns in clinical practice. The interactions occur firstly in the intestinal absorption for orally administered drugs. Aspirin and *Panax notoginseng* saponins (PNS)-based drugs are often combined in China to prevent larger-artery atherosclerosis. Here, we aimed to characterize the aspirin transport across Caco-2 cell monolayers, a model of the intestinal absorption, and further to evaluate the influence of PNS on aspirin hydrolysis and the relating mechanisms. Transcellular transport of aspirin and the influence of PNS were explored using Caco-2 cell monolayers. The protein expression of human carboxylesterase 1 (hCE1) and hCE2 in Caco-2 cells after PNS treatment was analyzed by ELISA, and the mRNA level were determined by qRT-PCR. In the study, Caco-2 cells showed high level of hydrolase activity, and most aspirin was hydrolyzed inside the cells during the transport process. Interestingly, PNS were demonstrated to inhibit the esterase activities responsible for aspirin hydrolysis in Caco-2 cells. PNS could also decrease the protein expression of hCE1 and hCE2, whereas exhibited minor effect on the mRNA expression. These results indicated that oral administration of PNS-based drugs might inhibit the hydrolysis of aspirin during intestinal absorption thus promoting its bioavailability.

## 1. Introduction

Oral administration is the most common drug delivery route because of the convenience and good patient compliance [1]. To reach the specific protein target in the human body, a drug is generally absorbed in the intestine and then enters into blood circulation after passing through the portal vein. To achieve this, a drug has to travel through several cell membranes such as small intestinal epithelial cells [2]. Caco-2 cell line is originated from human colonic adenocarcinoma and its morphology and biochemical functions are similar to the enterocytes. Caco-2 cells show differentiated brush borders with microvilli on the apical surfaces and the tight junctions including several transmembranes and intracellular proteins such as occludin, claudins and zona occludens [3]. Caco-2 cells also express some transporter proteins and metabolic enzymes, such as cytochrome P450 and carboxylesterases, which are responsible for the first-pass metabolism [4,5]. Owing to the similarity of their structure and functions with the small intestine, the Caco-2 cells model is widely used as a predictive tool to estimate intestinal absorption and drug-drug interactions [6,7,8].

**Figure 1:**
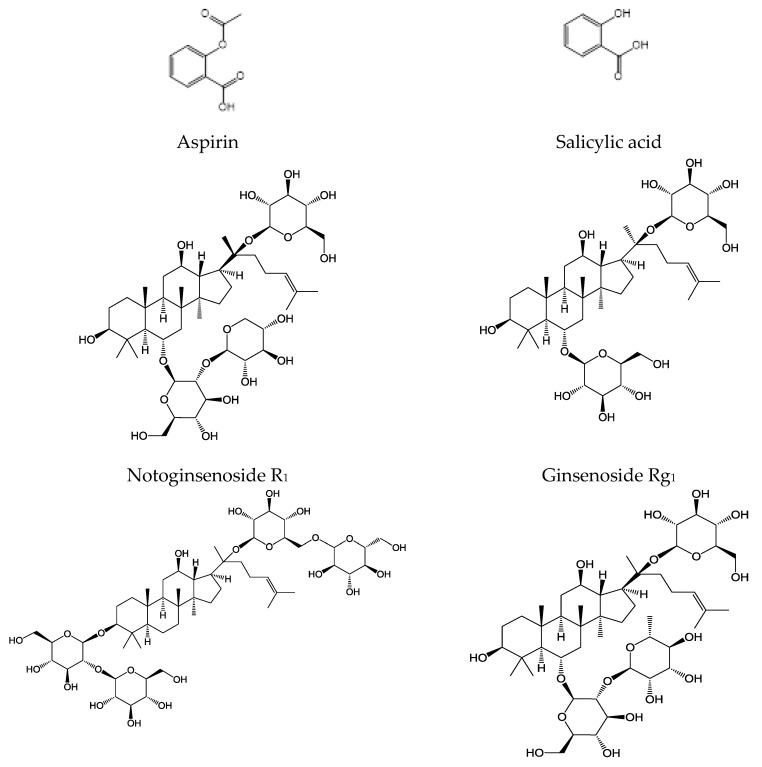

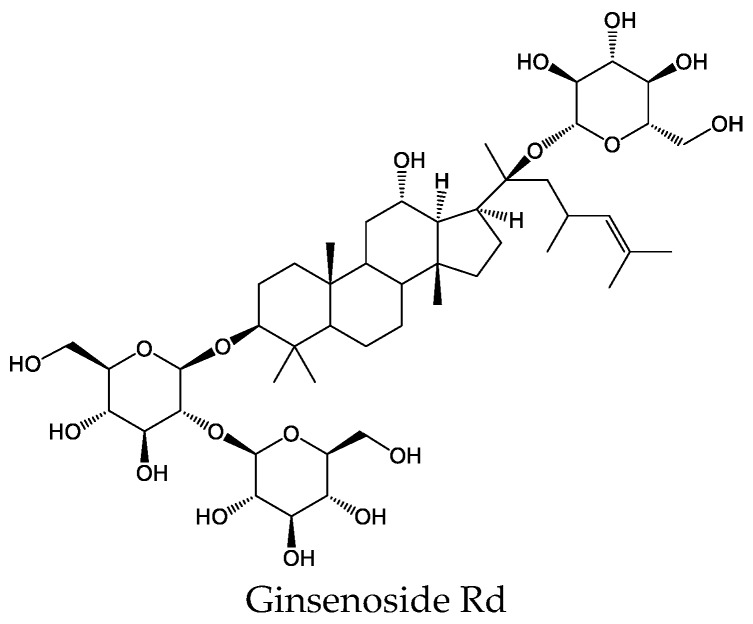
Chemical structures of aspirin, salicylic acid, notoginsenoside R_1_ and ginsenoside Rg_1_, Rb_1_, Re, Rd.

Aspirin (Figure 1) has been applied in clinical practice for more than a century and is still the most prescribed anti-platelet drug for primary prevention of cardiovascular disease and the secondary prevention of recurrent ischemic vascular events [9,10]. *Panax notoginseng* saponins (PNS) are the major pharmacologically active ingredients of notoginseng [*Panax notoginseng* (Burk.) F.H.C hen], a kind of perennial herbaceous plants affiliated to the Araliacede [11]. PNS contain over 20 different compounds named R_1_, Rb_1_, Rg_1_, Re and Rd (Figure 1) [12]. Many PNS-based drugs, such as Xuesaitong Capsule, Xuesaitong Granules and Xueshuantong Injection, have been developed and received regulatory approval on the cardio-cerebrovascular ischemic diseases in China. Given the similar functions, aspirin and PNS-based drugs are often combined to prevent thrombus. Good results have been achieved in clinical practice when the two drugs were taken together. However, herb-drug interactions (HDI) have already gained ever-increasing concerns in health practice in recent years [13,14,15]. Some researchers have reported that the bioavailability of a chemical drug may differ in the presence of complex chemical constituents derived from herbal drugs [16,17,18].

The ester linkage in aspirin can be hydrolyzed to salicylic acid (Figure 1) by esterases. After oral administration, aspirin will undergo hydrolysis in the intestine, liver, and plasma [19]. A group of specific esterases play a role in the hydrolysis in different tissues. For instance, human carboxylesterases (hCEs) are the major contributors to this activity in the liver and intestine [20,21,22].

Interestingly, the expression level of hCEs is high in Caco-2 cells as well as in the human intestine [4]. In our early research using MDCK-MDR1 cell model, it was found that the transport permeability of aspirin was greatly promoted in the presence of PNS [23]. However, an intensive investigation of the transport mechanism of aspirin combined with PNS across the intestinal cell monolayer has not yet been reported. In this work, we chose Caco-2 cells to illustrate the permeability of aspirin accompanied by enzymatic hydrolysis in the presence of PNS and the inhibitory influence of PNS on the esterases, especially hCEs.

## 2. Results

### 2.1. Cytotoxicology of Drugs in the Caco-2 Cell Line

**Figure 2:**
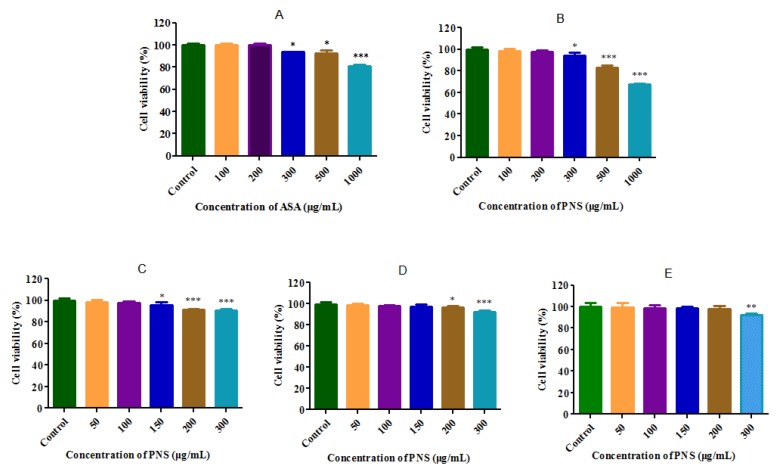
Cytotoxicity tests of aspirin (ASA), *Panax notoginseng* saponins (PNS) and their combinations as performed by MTT assay in Caco-2 cells. ASA and PNS had no cell cytotoxity under the concentration of 200 μg/mL, which was shown in (**A**) and (**B**). AP groups (PNS: ASA, 1:1, 3:2, 3:1, *w*/*w*) had no cell cytotoxity under the concentrations of 100 μg/mL, 150 μg/mL and 200 μg/mL in order, calculated by PNS, as shown in (**C**), (**D**), (**E**), respectively. Data were presented as mean ± SD (*n* = 5). *, ** and *** denoted results significantly different from those of the control group (*p* < 0.05, *p* < 0.01, and *p* < 0.001, respectively).

**Figure 3:**
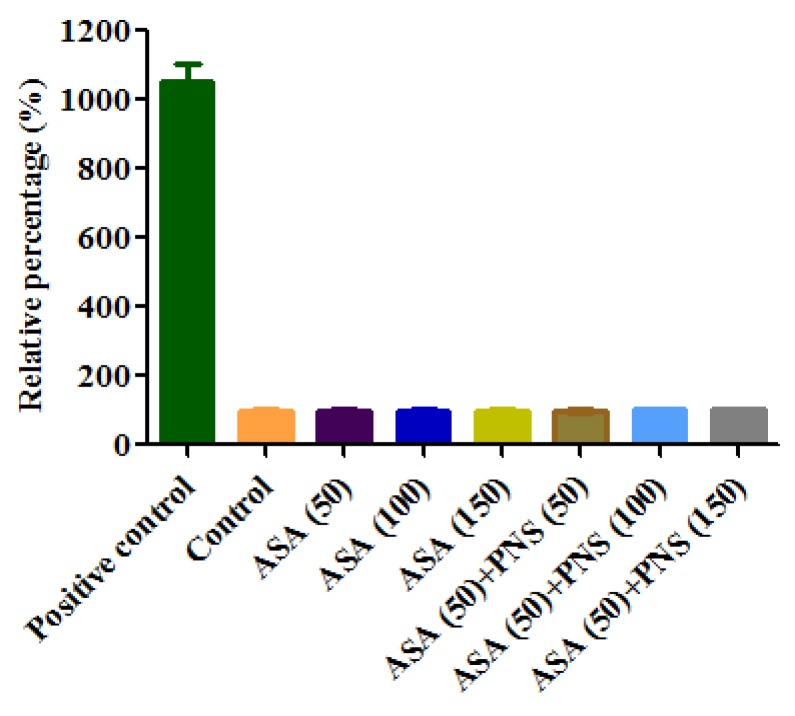
The cell membrane integrity of aspirin (ASA) or its combination with Panax notoginseng saponins (PNS) as detected by lactate dehydrogenase release assay. Compared with the control (untreated by drugs), ASA (50–150 μg/mL) had no considerable effect on integrity of cell membrane, and similar results were found in the combinations of ASA (50 μg/mL) and PNS (50–150 μg/mL). Data were presented as mean ± SD (*n* = 3).

Cell viability was conducted by an MTT assay to set the concentrations to be used in the following investigation, using the medium-treated cells group as control. As shown in Figure 2, aspirin or PNS exhibited no significant impact on cell viability in Caco-2 cells under the concentration of 200 μg/mL. In detail, AP groups (aspirin: PNS, 1:1, 1:2, and 1:3, *w*/*w*) showed no cytotoxicity within the concentration limit of 0–100 μg/mL, 0–150 μg/mL, and 0–200 mg/mL calculated by PNS, respectively. Figure 3 shows the effect of aspirin or its combination with PNS on cell membrane integrity by lactate dehydrogenase (LDH) release assay. The LDH release solution could lead to the loss of cell membrane in the positive control, resulting in a large amount of LDH poured into the cell culture. In contrast, no considerable effect on tested cells was observed for aspirin or its combinations with PNS at the tested concentrations.

### 2.2. Transport of Aspirin across Monolayers after PNS Addition

The aspirin transport across Caco-2 cells was investigated at concentrations of 50, 100 and 150 μg/mL. The results were presented in Table 1. The P_app_ value of apical (A) to basolateral (B) side for aspirin was close to 1 × 10^−^^6^ cm/s in the Caco-2 cell model. It appeared that aspirin transport had no significant difference in both directions (A→B or B→A) in the cell monolayers. The efflux ratio for aspirin at different concentrations was less than 1.5 in the cell model. 

**Figure 4:**
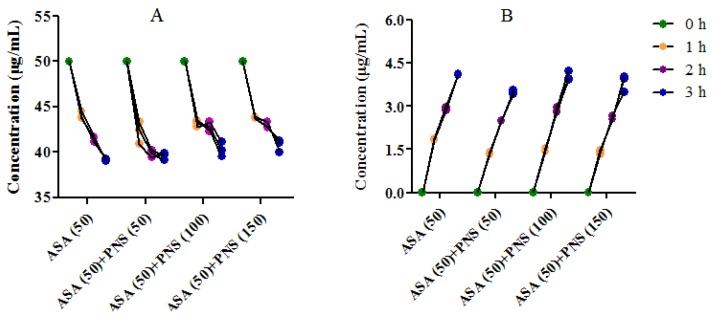
Aspirin (ASA) hydrolysis in apical (**A**) side of Caco-2 cell monolayers in the presence or absence of Panax notoginseng saponins (PNS). Washed Caco-2 cell monolayers were pre-incubated for 20 min in HBSS. ASA (50 μg/mL) or its combination with PNS in HBSS was loaded on A side, and HBSS was loaded on B side. (**A**) The amount of ASA in A side across Caco-2 cell monolayers over time (*n* = 3). (**B**) Salicylic acid in A side of ASA across Caco-2 cell monolayers over time (*n* = 3). Samples were collected at 1, 2 and 3 h from A side after treatment.

The concentrations of aspirin and salicylic acid in A side in the presence of PNS at different time were illustrated in Figure 4. In Caco-2 cells, aspirin was moderately hydrolyzed and rising concentrations of salicylic acid was observed in the A side. The ration of the amount of aspirin and salicylic acid in A side at 3 h showed that approximately 11% of total aspirin was hydrolyzed. Similarly, there was the same proportion of hydrolysis when aspirin (50 μg/mL) in the HBSS was placed at 37 °C for 3 h. This indicated that aspirin in the A side is only involved in natural hydrolysis without the involvement of esterases. The hydrolysis trends of aspirin in the A side show no significant changes in the presence of PNS (50–150 μg/mL), which indicates that PNS hardly affected the natural hydrolysis of aspirin.

**Figure 5:**
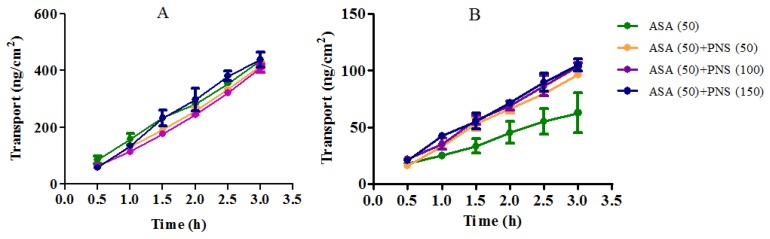
Effects of Panax notoginseng saponins (PNS) on aspirin (ASA) transport across Caco-2 cell monolayers from apical (**A**) to basolateral (**B**) side. Washed Caco-2 cell monolayers were pre-incubated for 20 min in HBSS. ASA (50 μg/mL) in HBSS with different concentrations of PNS was loaded on A side, and blank HBSS was loaded on B side. (**A**) Cumulative transport amount of ASA and ASA deduced from converted salicylic acid from A→B transport. (**B**) Cumulative transport amount of ASA from A→B transport. Samples were collected at 0.5, 1.0, 1.5, 2.0, 2.5 and 3.0 h from B side. Data were presented as mean ± SD (*n* = 3).

The influence of PNS on aspirin transport across Caco-2 cell monolayers was also investigated. As shown in Figure 5, the effects of PNS were studied at the concentrations of 50, 100 and 150 μg/mL in the presence of aspirin (50 μg/mL). In the B side, the amount of all permeated aspirin (aspirin together with salicylic acid (hydrolysed metabolite) increased gradually with time across the cell monolayers (Figure 5A). 

It appeared that there was no striking difference in comparison with the control (aspirin 50 μg/mL). However, compared to the control (aspirin 50 μg/mL), PNS treatment obviously increased the amount of aspirin across the cell monolayer (Figure 5B). The ration of aspirin and all permeated aspirin after the addition of PNS at 3 h were over 22%, higher than that of the control (aspirin 50 μg/mL) of 12%. Taken together, these data suggested that the aspirin hydrolysis mediated by esterases was inhibited by PNS.

**Figure 6:**
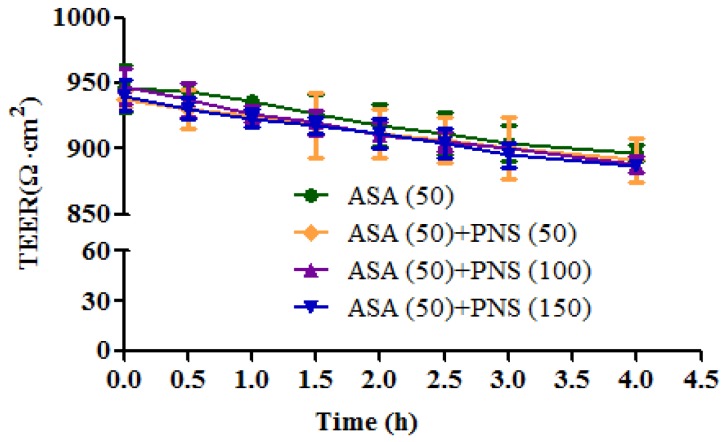
Transepithelial electrical resistance of Caco-2 cell monolayers monitored in the whole transport process from apical to basolateral direction. Resistance was measured using a Millcell-ERS resistance system and calculated from the background-corrected resistance. The TEER at each time point was presented as mean ± SD (*n* = 3).

In parallel to the transport experiment, the transepithelial electrical resistance (TEER) was measured in barrier function assays to investigate the changes of tight junctions in Caco-2 cells. As shown in Figure 6, aspirin (50 μg/mL) combined with PNS showed no visible difference relative to the control group (aspirin 50 μg/mL) regarding the trend of TEER changes. The observations indicated that the paracellular pathway was not involved in the transport of aspirin across the cell monolayers.

### 2.3. Aspirin Hydrolysis after PNS Addition

**Figure 7:**
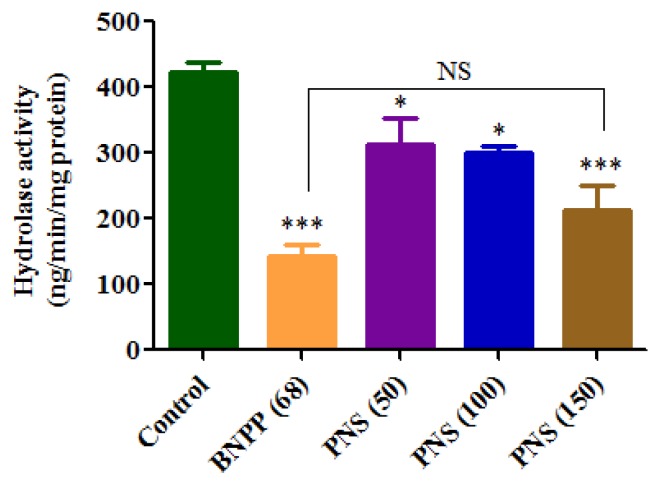
Hydrolysis of aspirin (ASA) in Caco-2 cells homogenates treated with various concentrations of Panax notoginseng saponins (PNS). Cell homogenates were prepared from Caco-2 cell monolayers and then diluted with 50 mM HEPES buffer (pH 7.4) at 1.2 mg/mL. Hydrolysis of ASA (1.8 μg/mL) in cell homogenates were carried out in the presence of PNS. No significance (NS) was detected between BNPP (68 μg/mL) and PNS (150 μg/mL). Data were presented as mean ± SD (*n* = 3). *, and *** denoted results significantly different from that of the control group (*p* < 0.05, *p* < 0.001).

The property of the esterases responsible for the hydrolysis of aspirin in Caco-2 cells was investigated in an inhibition experiment. The results were presented in Figure 7. The hydrolase activity was significantly inhibited by the addition of the bis-*p*-nitrophenyl phosphate (BNPP), a specific inhibitor of hCEs. 

Interestingly, PNS treatment significantly reduced the hydrolase activity in a concentration-dependent manner. As for the inhibitory effect, there was no significant difference between BNPP (68 μg/mL) and PNS (150 μg/mL). The findings suggested that PNS exhibited a potent efficiency in inhibiting the hydrolysis of aspirin. 

### 2.4. Inhibitory Potential of PNS on hCE1 and hCE2

**Figure 8:**
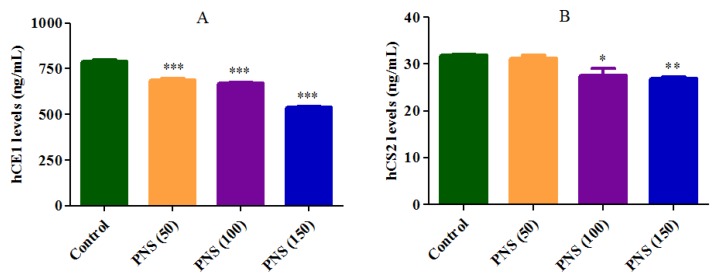
Effects on human carboxylesterase 1 (hCE1) (**A**) and hCE2 (**B**) protein level following Panax notoginseng saponins (PNS) treatment. Cells were incubated with PNS for up to 24 h. After the removal of PNS, cell lysates were prepared for ELISA analysis. Data were presented as mean ± SD (*n* = 3). *, **, and *** denoted results significantly different from those of the control group (*p* < 0.05, *p* < 0.01, and *p* < 0.001, respectively).

**Figure 9:**
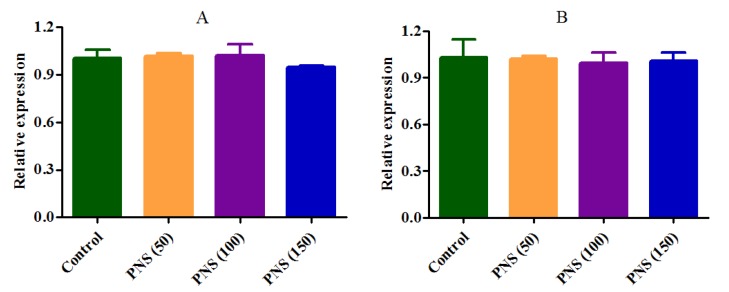
Effects on mRNA expression of human carboxylesterase 1 (hCE1) (**A**) and hCE2 (**B**) following Panax notoginseng saponins (PNS) treatment. Cells were incubated with PNS for up to 24 h. After the removal of PNS, total mRNA was prepared for qRT-PCR. Data were presented as mean ± SD (*n* = 3).

The Caco-2 cell lysates were analyzed for hCE1 and hCE2 protein by ELISA. After 24 h incubation, PNS (50, 100, 150 μg/mL) significantly decreased the hCE1 protein level in a concentration-dependent manner, compared to the control (Figure 8A). Meanwhile, the hCE2 protein levels were also significantly inhibited by PNS (100, 150 μg/mL) (Figure 8B). On the other hand, there were no significant difference on the mRNA expression of hCE1 and hCE2 following treatment with PNS (Figure 9). Collectively, for hCE1 and hCE2, our data indicated that PNS treatment mainly down-regulated the expression level of the protein rather than the mRNA.

## 3. Discussion

Almost all herbs are orally administrated. The intestinal tract presents various chemical and enzymatic barriers that affect the oral delivery of drugs [24]. Caco-2 cells are considered to be the most common in vitro model for human intestinal absorption and metabolism [25,26]. Living cells can reduce MTT to formazan, while dead cells cannot [27]. The cell viability after exposure to different drugs may be evaluated by measuring MTT metabolism. The LDH release method is widely used to detect the cell membrane integrity. The concentrations that showed no cytotoxicity were used for further experiments in the study. 

The present study showed that the transepithelial fluxes of aspirin in both directions did not differ significantly. The efflux ratio of aspirin was less than 1.5 in the cell model. Aspirin transport across the cell monolayers increased with an increase in concentration. These results indicated that passive diffusion might be the main pattern, which is consistent with our previous report [23]. The permeability coefficients may vary among different cell lines due to their physiological properties such as the formation of tight junctions. 

Aspirin is often used in clinical practice combined with PNS-based drugs. We found that aspirin had undergone abundant hydrolysis involved with esterases inside Caco-2 cells. Interestingly, aspirin hydrolysis was obviously inhibited by PNS treatment though total fluxes of aspirin hardly altered. Caco-2 cells can express esterases that are present in the human enterocyte [4]. Ester-containing drugs across the intestinal epithelium are limited by esterases such as hCEs [28,29]. Hence, such inhibition of aspirin hydrolysis exerted by PNS may also be present in human epithelium cells.

The measurement of TEER is an easy and quick method to estimate the tight junction integrity [30]. Generally, the decrease in TEER during the transport experiment could indicate opening of tight junctions, thus facilitating drug permeation. The Caco-2 cell monolayers were considered as qualified when the TEER reached above 600 Ω·cm^2^ [31]. In this study, the TEER value among groups ranged from 900–950 Ω·cm^2^ throughout the transport process. The fact that PNS treatment caused little changes on the tight junction integrity could be suitable to explain that minor alternations of PNS on the permeated fluxes of aspirin via cell monolayers here.

To provide more insight into the influence of PNS on aspirin hydrolysis inside Caco-2 cells, an inhibition experiment was conducted in the cell homogenates. The present finding showed that aspirin hydrolysis was not completely inhibited in the presence of BNPP. We hypothesized that the hCEs-mediated hydrolysis of aspirin had already been completely inhibited. So, the results indicated that apart from hCEs, there might be other esterases responsible for the hydrolysis of aspirin. Importantly, PNS treatment had exhibited concentration-dependent inhibitory effect on the activity of esterases and hence indirectly rose up the level of aspirin. To our knowledge, it is the first report about the inhibition of esterases treated by PNS in Caco-2 cells. In a previous study, hCEs had been shown to be involved in aspirin hydrolysis [22]. Recent reports have revealed that many natural triterpenoids were found to display the inhibitory effects on hCEs [32,33,34]. A Master dissertation also reported that gensenosides exhibited the inhibition effect toward hCEs [35]. PNS are the multi-component system with triterpenoids as major constituents containing a group of ginsenosides. Hence, we speculated that PNS owned the inhibitory activity against hCEs. Whereas, the inhibitory effect of PNS must be complicated due to a combined influence of many compounds. More studies are under way to clarify the inhibitory mechanism of PNS on hCEs and other drug-metabolizing enzymes in our lab. 

We further investigated the inhibitory potential of PNS on hCEs, the major metabolic esterases in human intestine [3]. The decreased protein level of hCE1 and hCE2 in Caco-2 cells observed after PNS treatment indicated that the interactions were not just simple inhibition. In contrast, the treatment did not decrease the level of mRNA expression of hCE1 and hCE2. Taken together, we speculated that the inhibitory influence of PNS might result from the reduced translation or the increased degradation of hCE1 and hCE2. It should be point out that human small intestine mainly contains hCE2, with smaller quantities of hCE1, which is quite different in Caco-2 cells [36]. Moreover, hCE1 and hCE2 show different substrate specificities. Aspirin is predominately hydrolyzed by hCE2, and slightly hydrolyzed by hCE1 [22]. Therefore, the study is not a comprehensive description about the prediction of human intestinal absorption of aspirin, and more research is required.

## 4. Materials and Methods 

### 4.1. Materials

Aspirin was obtained from the National Institute for the Control of Pharmaceutical and Biological Products (Beijing, China). PNS was obtained from Yunnan Sanqi Technology Co., Ltd. (Wenshan, China). PNS content was determined as: notoginsenoside R_1_, 7.4%; ginsenoside Rg_1_, 26.3%; ginsenoside Rb_1_, 27.7%; ginsenoside Re, 3.7%; ginsenoside Rd, 7.6%. Minimum essential medium (MEM), fetal bovine serum (FBS), non-essential amino acids (NEAA), penicillin, streptomycin, and 0.05% trypsin-EDTA solution were obtained from Gibco (Thermo Fisher Scientific Co., Waltham, MA, USA). All other chemicals and solvents used were analytical or chromatographic grade.

### 4.2. Cell Culture

Caco-2 cells were obtained from the National Infrastructure of Cell Line Resources (Beijing, China). The cells were grown in culture flasks using MEM supplemented with 10% (*v/v*) FBS, 1% (*v/v*) NEAA and 1% (*v/v*) antibiotic solutions (100 U/mL penicillin and 100 μg/L streptomycin). The cells were grown in a controlled atmosphere of 5% CO_2_ and 90% relative humidity at 37 °C. On reaching 80%–90% confluence, the cells were subcultured at a 1:3 split ratio using 0.05% trypsin. For the study, cells between passage 30~40 were used.

### 4.3. Cytotoxicology of Drugs in the Caco-2 Cell Line

The cell viability was examined by an MTT assay. Briefly, 1 × 10^4^ cells per well were seeded in 96-well plates. Following 48 h of culture, the cells were exposed to a series of concentrations of aspirin or PNS and their combinations for 24 h. The cells were then incubated with 20 μL MTT solution (5 mg/mL) for 4 h. At the end of incubation, MTT solution was discarded and the formazan crystals were completely dissolved using 150 μL DMSO per well. The plates were gently shaken for 5 min and measured at 490 nm using an automatic microplate reader (Multiskan Go, Thermo Fisher Scientific Co., Waltham, MA, USA). All MTT assays were performed using five separate trials. The cell viability of the untreated control was arbitrarily considered as 100%.

The impact of drugs on the cell membrane was detected using a LDH assay kit (BeyGEN, Nanjing, China). Briefly, the Caco-2 cells cultured in a 96-well plate were incubated with a serum-free MEM containing aspirin or its combination with PNS at 37 °C for 4 h. After that, 20 μL of LDH release solution was added into the positive control and incubated for 1 h. Subsequently, 120 μL of the obtained cell supernatant was transferred to another 96-well plate. After being treated according to the manufacture’s protocols, the absorbance at 490 nm was immediately recorded using an automatic microplate reader. Cells incubated with serum-free MEM alone were used as comparison.

### 4.4. Transport of Aspirin across Monolayers after PNS Addition

The impact of aspirin absorption after PNS addition was investigated using our previously published method [37]. Briefly, Caco-2 cells were seeded at 2 × 10^5^ cells/mL on the PET membranes in transwell chambers and allowed to form monolayers. Before the experiment, the cells were gently washed three times with HBSS and pre-incubated for 20 min at 37 °C. Approximately, Caco-2 TEER values exceed 900 Ω·cm^2^. The drug solution (0.5 mL) was added in A side, and HBSS (1.5 mL) was added in B side to measure A→B transport. The B→A transport was assessed by adding 1.5 mL HBSS of drug solution in B side and 0.5 mL HBSS in A side. The cells were shaken (50 rpm) at 37 °C. Samples (600 μL) were withdrawn from B side (to evaluate A→B) and 200 μL of samples was withdrawn from A side (to evaluate B→A) at different time intervals. Samples were rapidly mixed with acetic acid (final concentration 2%) and then centrifuged at 10,000 rpm for 10 min at 4 °C. The supernatants were analyzed by HPLC.

During the experiment, The TEER value of the untreated cells and the cells treated with aspirin or its combinations with PNS was measured using a Millipore Millicell ERS system equipped with chopstick electrodes (Millipore Corporation, Billerica, MA, USA). An insert that did not contain cells was used as background resistance. TEER was calculated from the background-corrected resistance.

### 4.5. Aspirin Hydrolysis after PNS Addition

The changes of aspirin hydrolysis in Caco-2 cell homogenates after the addition of PNS was investigated using a previously published method with some modifications [38]. The cell monolayers were washed twice with ice-cold PBS and then detached with a spatula. The harvested cells were suspended in ice-cold buffer (292 mM sucrose, 1 mM EDTA, and 50 mM Tris) and then homogenized using an ultrasonic homogenizer (SCIENTZ-IID, Ningbo, China) under the ice-cold condition. The protein content of the homogenates was determined by BCA Protein Assay Kit (KeyGen BioTECH, Nanjing, China) using bovine serum albumin as a standard. 

The homogenates of Caco-2 cells were diluted with HEPES buffer (50 mM, pH 7.4, to the appropriate protein concentrations. After pre-incubation for 5 min at 37 °C, dimethyl sulfoxide (DMSO, control), BNPP or PNS dissolved in DMSO, were added to the reaction solution and pre-incubated for 5 min. The reaction was then started by adding aspirin dissolved in HEPES buffer. The final concentration of DMSO in the hydrolyzing incubation system was maintained at 1.0%, which had no effect on hydrolase activity. The reaction was terminated by adding an equal volume of ice-cold methanol. After centrifugation of the reaction mixture at 6000 rpm for 10 min, 200 μL of the supernatants was mixed with acetic acid (final concentration of 2%) and analyzed by HPLC.

### 4.6. ELISA Analysis for hCE1 and hCE2

The expression level of hCE1 or hCE2 in Caco-2 cells treated by PNS was quantitatively measured using hCEs ELISA Kit (RayBiotech, Norcross, GA, USA) according to the manufacturer’s protocol. Briefly, 2 × 10^5^ cells were seeded in each well of a 6-well plate for 24 h and then incubated with different concentrations of PNS at 37 °C for another 24 h. The cells (the untreated cells or the cells treated with PNS) were washed with cold PBS for three times. Total cellular protein was extracted with RIPA lysis buffer (KeyGen BioTECH) and protease inhibitor mixture. Then, the cell lysis buffer was re-suspended and centrifuged at 10,000 rpm for 5 min. The supernatants were collected and assayed. The absorbance was detected at 450 nm using an automatic microplate reader.

### 4.7. qRT-PCR for hCE1 and hCE2

Total RNA was harvested from the treated or untreated cells using the EasyPure^®^ RNA kit (Transgen-Biotech, Beijing, China), as recommended by the manufacturer. Total RNA was reversely transcripted using the TransScript^®^ One-Step gDNA Removal and cDNA Synthesis SuperMix (Transgen-Biotech). Genes of interest were amplified using TransScript^®^ Top Green qPCR SuperMix. Reactions were initiated at 95 °C for 3 min, followed by 45 cycles of 95 °C for 30 s, 65 °C for 30 s, and 72 °C for 60 s. Melting curve analysis was performed in order to confirm specificity of primers. The relative mRNA expression was normalized to GAPDH and calculated using the 2^−ΔΔCt^ method.

The following primers were used for qRT-PCR:

hCE1-F, AGAGGAGCTCTTGGAGACGACAT;
hCE1-R, ACTCCTGCTTGTTAATTCCGACC;
hCE2-F, CCATGGTGATGAGCTTCCTTTTGT;
hCE2-R, AGGTATTGCTCCTCCTGGTCGAA;
GAPDH-F, CTCCTCCACCTTTGACGCTG;
GAPDH-R, TCCTCTTGTGCTCTTGCTGG.

### 4.8. HPLC Analysis

The experiment samples were analyzed on an Agilent 1200 system (Agilent Technologies Inc., Santa Clara, CA, USA) equipped with a VWD detector. The analytical column was a Kromasil^®^ C_18_ column (4.6 mm × 250 mm, 5 μm). The mobile phase consisted of acetonitrile, water and acetic acid with the ratio of 29:69:2. The flow rate was delivered at 1 mL/min. An injection volume of 100 μL was used. Aspirin and salicylic acid were detected at wavelengths of 276 nm and 303 nm, respectively.

### 4.9. Statistical Analysis

The apparent permeability coefficients (P_app_) for aspirin were calculated by the following equation:

(1) $$P_{\mathrm{app}}\;=\frac{dQ/dt}{SC}$$

where *dQ/dt* is the apparent appearance rate of aspirin in the receiver side; S is the surface area of monolayers, and C is the initial aspirin concentration in the donor chamber. Data were represented as mean ± SD from at least three independent measurements. Statistical analysis was performed with ANOVA, and *p* < 0.05 was considered as statistically significant (* *p* < 0.05, ** *p* < 0.01, and *** *p* < 0.001).

## 5. Conclusions

The present work demonstrated that Caco-2 cells show high levels of hydrolase activity during aspirin transport. Importantly, PNS were found to inhibit the esterase activities responsible for aspirin hydrolysis, resulting in an improved aspirin level across Caco-2 cells. Further, PNS decreased the protein expression of hCE1 and hCE2, though it exhibited minor effects on the mRNA expression. It is hoped that these findings will help to provide information on the underlying safety of concomitant use of aspirin and PNS-based drugs in clinical practice.

## Figures and Tables

**Table 1:** Various concentrations of aspirin transport across Caco-2 cell monolayers.

Condition	P_app_ (A→B) ± SD (× 10^−^^6^ cm/s)	P_app_ (B→A) ± SD (× 10^−^^6^ cm/s)	Efflux Ratio (B→A)/(A→B)
50 μg/mL	0.98 ± 0.07	1.10 ± 0.08	1.12
100 μg/mL	0.98 ± 0.01	1.08 ± 0.09	1.10
150 μg/mL	0.96 ± 0.05	1.02 ± 0.08	1.06

P_app_, apparent permeability; A, apical side; B, basolateral side. Values are mean ± SD (*n* = 3). P_app_ for aspirin was calculated by the sum of aspirin together with salicylic acid (hydrolysed metabolite).
